# The Effect of Maximal Strength Training on Strength, Walking, and Balance in People with Multiple Sclerosis: A Pilot Study

**DOI:** 10.1155/2016/5235971

**Published:** 2016-12-26

**Authors:** Herb I. Karpatkin, Evan T. Cohen, Sarah Klein, David Park, Charles Wright, Michael Zervas

**Affiliations:** ^1^Department of Physical Therapy, Hunter College, City University of New York, 425 East 25th Street, New York, NY, USA; ^2^Department of Rehabilitation and Movement Sciences, School of Health Professions, Rutgers, The State University of New Jersey, 40 East Laurel Road, Suite 2105, Stratford, NJ 08084, USA

## Abstract

There is little literature examining the use of maximal strength training (MST) in people with multiple sclerosis (pwMS). This pretest-posttest study examined the effects of a MST program on strength, walking, balance, and fatigue in a sample of pwMS. Seven pwMS (median EDSS 3.0, IQR 1.5) participated in a MST program twice weekly for eight weeks. Strength was assessed with 1-repetition maximum (1RM) on each leg. Walking and balance were measured with the 6-Minute Walk Test (6MWT) and Berg Balance Scale (BBS), respectively. Fatigue was measured during each week of the program with the Fatigue Severity Scale (FSS). The program was well tolerated, with an attendance rate of 96.4%. Participants had significant improvements in right leg 1RM (*t*(6) = −6.032, *P* = 0.001), left leg 1RM (*t*(6) = −5.388, *P* = 0.002), 6MWT distance (*t*(6) = −2.572, *P* = 0.042), and BBS score (*Z* = −2.371, *P* = 0.018) after the MST intervention. There was no significant change in FSS scores (*F*(1, 3.312) = 2.411, *P* = 0.092). Participants in the MST program experienced improved balance and walking without an increase in fatigue. This MST program may be utilized by rehabilitation clinicians to improve lower extremity strength, balance, and mobility in pwMS.

## 1. Introduction

Multiple sclerosis (MS) is a progressive neurologic disorder that often results in impaired mobility. The effects of resistance training in ameliorating mobility deficits in people with MS (pwMS) have been previously studied, but with inconsistent methodologies. White et al. found improvements in 25-foot walk test performance and self-reported fatigue in a sample of pwMS following a twice weekly, 8-week-long lower extremity strengthening program at 70% of the participants' maximal voluntary contraction (MVC) [1]. Taylor et al. used a twice weekly, 10-week-long program of combined upper and lower body exercises at 60–80% of participants' one-repetition maximum (1RM, i.e., the maximum amount of weight a person can lift in a single repetition of an exercise) and found improvements in walking speed and a trend towards improvement in the 2-Minute Walk Test [2]. Gutierrez et al. used a twice weekly, 8-week-long program where participants performed lower extremity strengthening at 70% of their 1RM [3]. Improvements were seen in gait kinematics and in endurance measured by a 3-minute stepping task. In a systematic review of resistance training for pwMS, Kjølhede et al. found strong evidence to support the use of progressive resistive training for improving muscle strength, while evidence for its effects on functional mobility and fatigue was less strong [4].

A common finding in the literature on resistance training programs for pwMS is that they have exclusively utilized relatively mild to moderate resistance loads, rarely exceeding more than 70% of the 1RM or MVC. Although no specific rationales were provided, it could be conjectured that use of heavier training loads was discouraged to limit complications of neurogenic fatigue. Another limitation is that the shorter duration walking tests (i.e., the 2-Minute Walk Test) used as outcome measures in these studies may not have been sufficiently sensitive to change, particularly in those with less MS-related disability [5].

In contrast to the training programs where mild to moderate resistance is used, maximal strength training (MST), where the loads are 85–95% of the person's 1RM, is found sparingly in the rehabilitation literature. Hill et al. found small but significant improvements in 6-Minute Walk Test (6MWT) distance in a sample of people with chronic stroke after performing a lower extremity MST program three times per week for eight weeks [6]. Other measures of mobility were unchanged. Jayaraman et al. used a MST program for 12 sessions over 4 weeks on a sample of people with incomplete spinal cord injury and found improvements in the Berg Balance Scale (BBS), 6MWT, and measures of spasticity [7]. Fimland et al. examined the use of MST in a sample of pwMS using unilateral leg press (i.e., hip and knee extension) and ankle plantarflexion exercises at 85–90% of participants' 1RM 5 times per week for 3 weeks [8]. Although mobility outcomes were not measured, there was EMG evidence of increased efferent neural outflow, suggesting that MST may have resulted in increased central activation.

These studies suggest that MST is well tolerated by persons with neurologic pathology, can improve functional measures, and may facilitate neuromotor adaptations. However, the effects of MST on mobility measures in pwMS have not been examined. The main purpose of this study is to examine the effects a lower extremity MST program on walking endurance and balance in a sample of pwMS. Due the intensive nature of MST and the common finding of fatigue in MS, the secondary purpose is to examine the effects of MST training on fatigue. We hypothesized that pwMS will show improvements in walking endurance, balance, and strength following a lower extremity MST. We also hypothesized that perceived fatigue would not increase because of the MST training. If these hypotheses are correct, it suggests that clinicians who treat pwMS could safely and effectively utilize MST in their rehabilitation programs.

## 2. Methods

This was a prospective, exploratory, pretest-posttest design pilot study. This study was approved by the relevant Institutional Review Board. All participants provided informed consent for their involvement in the study.

### 2.1. Participants

A convenience sample of eight pwMS were recruited from a local multidisciplinary MS specialty clinic. Participants needed to have a definitive diagnosis of MS, to be at least 18 years of age, and to be able to ambulate for 6 minutes, with or without an assistive device. Exclusion criteria included any type of orthopedic or cardiopulmonary condition restricting the ability to walk for 6 minutes and any MS exacerbations in the 2 weeks prior to the start of this study.

### 2.2. Procedures

Demographic and participant characteristic data collected included EDSS, years since MS diagnosis, age, and sex. MS-disease impact was measured with the Multiple Sclerosis Impact Scale (MSIS-29) [9]. Fatigue was measured with the Fatigue Severity Scale (FSS) [10], a widely used, valid and reliable measure of the impact of fatigue on participation in pwMS.

To determine 1RM, participants were first familiarized with the horizontal leg press machine and set up with their hips and knees aligned at 90-degree angles. Once in proper alignment, participants began with minimal weight while performing unilateral leg press extensions to gain comfort in performing the exercise. The load was then increased in 4.5 kg increments to a level that participants felt was about 50–75% of their maximum capacity. Single repetitions were performed with increasing weight (2.3–4.5 kg) until only 1 repetition could be completed. The weight at which the participants could perform a single repetition with each lower extremity was recorded as the 1RM. This protocol is recommended by the American College of Sports and Medicine [11] and has been previously utilized in a study of PwMS [8].

Balance was measured with the Berg Balance Scale (BBS). The BBS measures a person's ability to balance during 14 different tasks of varied difficulty. Items are rated by a scale from 0 to 4 and are summed to the total score which has a maximum (best) value of 56. The BBS is a valid and reliable test of balance in pwMS [12]. Walking endurance was measured with the 6MWT. The 6MWT measures the total distance walked for six minutes. The 6MWT is a valid and reliable test of walking endurance in PwMS [13].

### 2.3. Intervention

The MST intervention occurred twice weekly for eight weeks. Each MST session began with a 5-minute-long warm-up on a cycle ergometer without resistance at a participant-selected comfortable pace. This was followed by 5 minutes of seated rest. Participants then performed a warm-up of five repetitions on the horizontal leg press machine at 50% of their 1RM. Ninety seconds later, participants began the MST regimen which consisted of 4 sets of 4 repetitions at 85–95% of their 1RM for each leg, with a 90-second break between each set. Participants were instructed to perform a concentric contraction followed by a slower controlled eccentric contraction, such that the time spent was in a 1 : 2 time ratio, respectively. This timing model has been used in previous studies of MST (e.g., Mosti et al. [14]). Participants were instructed to pause a full second before performing the next repetition to avoid elastic energy contribution to the movement and instructed not to hold their breath. If a participant could perform all 4 sets of 4 repetitions at the designated weight and thought he/she could perform a fifth repetition, the weight was increased by 1.1–2.3 kg at the next session. The increase in resistance was determined by participant capability and clinical judgment of the researchers. At the end of each MST session, a cooldown was performed at a participant-selected comfortable pace on the cycle ergometer for five minutes.

### 2.4. Assessments

Demographic information was collected after entry into the study. Baseline testing included 1RM, BBS, 6MWT, MSIS-29, and FSS. FSS was also measured during each week to determine whether there was any impact of the MST program on fatigue during the intervention. At the end of the eight-week training program, participants again performed the 1RM test, the BBS, and the 6MWT.

### 2.5. Statistical Analyses

Analyses were performed with SPSS, version 23.0 (IBM, Inc., Chicago, IL). Pretest-Posttest comparisons were made with repeated-measures *t*-tests for 1RM and 6MWT and Wilcoxon Signed-Ranks Test for the BBS. All results were analyzed using the group means for each variable. FSS measures across the MST session were analyzed with a repeated-measures ANOVA. The a priori alpha level was set at <0.05.

## 3. Results

### 3.1. Participant Characteristics

Eight pwMS participated in the study. Seven completed the MST training protocol, while one participant dropped out due to an unrelated injury. The characteristics of the participants who completed the MST training protocol can be found in Table 1.

### 3.2. Attendance and Adherence

Four participants completed all 16 MST sessions, two completed 15 MST sessions, and one completed 14 sessions for an overall attendance rate of 96.4%.

### 3.3. 1-Repetition Maximum (1RM) Performance

**Figure 1:**
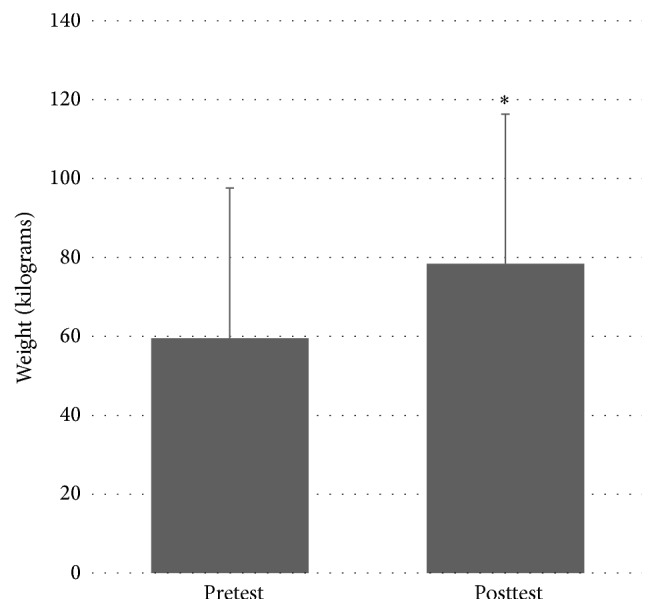
Pretest and posttest values for right leg 1RM. *∗* denotes statistically significant difference at *P* < 0.05.

**Figure 2:**
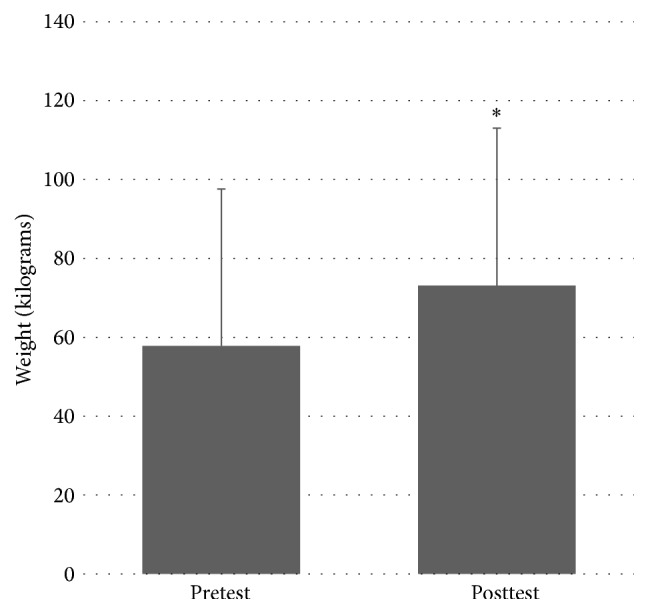
Pretest and posttest values for left leg 1RM. *∗* denotes statistically significant difference at *P* < 0.05.

There was a significant effect of MST on right leg 1RM weight (in kg), between pretest (*M* = 59.5, SD = 38.1) and posttest (*M* = 78.4, SD = 37.9); *t*(6) = −6.032, *P* = 0.001. There was also a significant effect of MST on left leg 1RM weight between pretest (*M* = 57.8, SD = 39.8) and posttest (*M* = 73.1, SD = 39.9); *t*(6) = −5.388, *P* = 0.002. These indicate that a significant increase in leg press strength was found in both lower extremities after the MST program. Please see Figures 1 and 2 for right and left leg results, respectively.

### 3.4. 6-Minute Walk Test Performance

**Figure 3:**
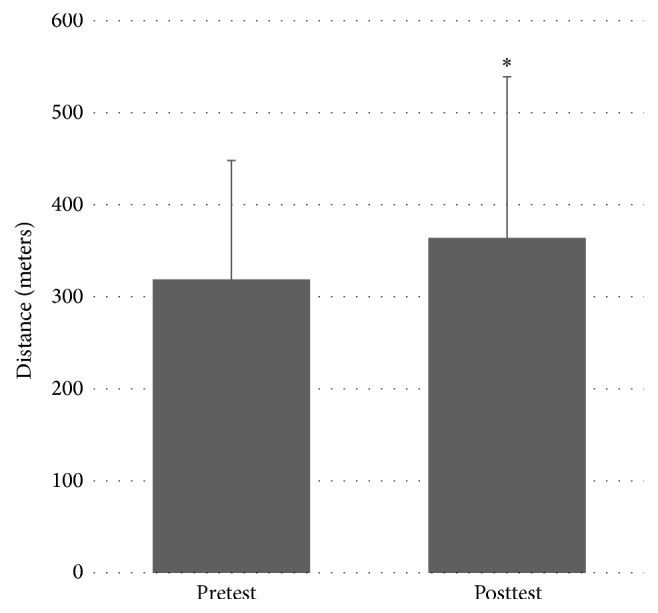
Pretest and posttest values for 6-Minute Walk Test distance. *∗* denotes statistically significant difference at *P* < 0.05.

There was a significant effect of MST on 6MWT distance (in meters) between pretest (*M* = 318.8, SD = 129.2) and posttest (*M* = 364.1, SD = 174.8); *t*(6) = −2.572, *P* = 0.042. This indicates an improvement in 6MWT distance after the MST program. Please see Figure 3.

### 3.5. Berg Balance Scale Score Performance

**Figure 4:**
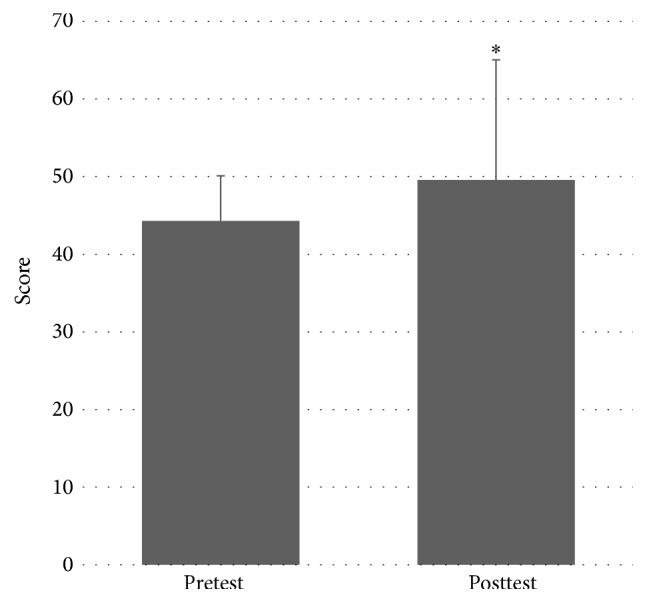
Pretest and posttest values for Berg Balance Scale scores. *∗* denotes statistically significant difference at *P* < 0.05.

There was a significant effect of MST on BBS score between pretest (median = 46, IQR 16) and posttest (median = 49, IQR 8); *Z* = −2.371, *P* = 0.018. This indicates an improvement in BBS distance after the MST program. Please see Figure 4.

### 3.6. Fatigue Severity Scale Scores

**Figure 5:**
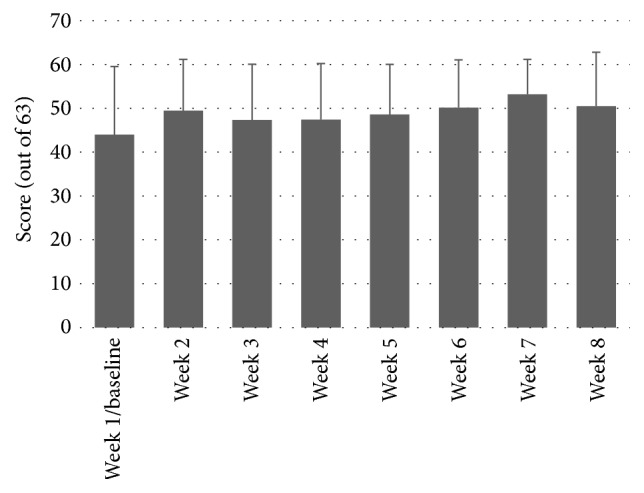
Week-by-week Fatigue Severity Scale scores.

Because Mauchly's Test of Sphericity was significant, the more conservative Greenhouse-Geisser corrected degrees of freedom were used for this ANOVA. There was no significant effect of MST on FSS throughout the MST protocol, *F*(1, 3.312) = 2.411, *P* = 0.092. This indicates that there was no change in the perceived impact of fatigue on participation through the duration of the study. Please see Figure 5. The lack of change in FSS scores coupled with high program adherence indicates that the program was well tolerated by the participants without inducing an increase in perceived fatigue.

## 4. Discussion

In this study, we examined the effect of lower extremity MST on functional mobility in a sample of pwMS. The results demonstrated that pwMS who underwent an 8-week lower extremity MST program experienced improvements in both balance (as evidenced by significant improvements in the BBS) and walking endurance (as evidenced by significant improvements in the 6MWT). In addition, the program was well tolerated as evidenced by its high compliance rate and absence of adverse events. Despite the intensive nature of the intervention and the prevalence of MS fatigue, the MST program did not result in a significant increase in fatigue (FSS). Interventions that are targeted at improving mobility measures in pwMS are important as mobility loss is one of the major issues leading to reduced quality of life in this population [15]. In particular, reduced walking endurance and increased risk of falls due to balance impairment are a frequent finding [16]. The results of this study provide evidence that MST may be a viable means of improving these problems in pwMS.

Limited walking endurance is another common problem in pwMS, but physical therapy interventions to improve this have been of limited success. The 6MWT is a critical measure for assessing walking endurance in the MS population as it involves walking for periods of time that approximate community ambulation. Participants in this study improved an average of 46.0 m, which strongly suggests improvements in walking endurance. This improvement is well outside the SEM of 27.48 meters established for this population [17], indicating that the improvements seen were unlikely to be due to measurement error.

The participants in this study did not receive any additional walking training outside of their normal daily activities. Therefore, walking endurance improved despite the absence of specific walking-oriented interventions.

Falls are a frequent occurrence in pwMS, occurring in over half of all persons diagnosed, with the risk worsening as disability increases [18]. The BBS is a valid [12] and reliable [13] measure for assessing falls risk and balance ability in pwMS. In this study, participants improved an average of 5.3 points on the BBS. This approaches but does not reach the minimal detectable change value of 7 [17]. The change does, however, exceed the SEM of 3 established for this population [17], suggesting that the score was unlikely to be due to measurement error. As with the improvements seen in walking endurance, the participants in this study did not receive any additional balance training during the study period. The results of this study represent an example of a series of specific tasks (e.g., items in the BBS) improving despite the absence of task specific training.

The fact that improvements in walking and balance measures occurred despite the absence of specific training for these measures is intriguing. Force production in the hip and knee extensors is certainly a factor in mobility, and improvements in these areas could help improve balance and walking endurance. However, baseline measures of strength taken for each participant did not reveal significant weakness in their hip or knee extensors prior to the training, so it is unlikely that the changes seen in the BBS and the 6MWT were due solely to increased force production. An alternative explanation for the improvement has been suggested by Fimland et al. [8], who showed that MST in pwMS resulted in increased neural drive. Neural drive has been defined as “the ensemble of action potentials from a group of alpha motor neurons to the innervated muscle” [19]. With strength training, the increases in neural drive can influence the activation of muscles through modulation or changes in motor unit recruitment, synchronization, firing frequency, and intermuscular coordination [20]. These neural changes are seen earlier in a strengthening protocol compared to the slower process of muscle hypertrophy. The central nervous system changes that occur in MS may result in decreased neural drive to structures that mediate walking and balance. The increases in neural drive following MST may have resulted in greater motor recruitment, synchronization, firing frequency, and intermuscular coordination which may have been responsible for the improvements seen in the BBS and 6MWT.

A concern for the participants in this study was that the MST may have been too aggressive for them leading to an increase in fatigue and thus further limit their mobility. Exercise-induced fatigue in pwMS has historically been a concern of clinicians [21]. Even though the notion that exercise can lead to worsening of mobility has been debunked [22], clinicians may still feel reluctant to use aggressive interventions such as MST. Despite the intensity of the intervention in this study, there was no significant worsening of fatigue as measured by the FSS. There was, however, a trend of increasing FSS scores over the course of the eight-week MST program. The mean FSS increase of 6/63 points does approach the SEM of 6.3/63 (extrapolated from the 0.7/9 reported by Learmonth and colleagues) [23] but does not approach the minimal detectable change of 17.1/63 (extrapolated from the 1.7/7 reported by Learmonth and colleagues). Given the small sample size and the variability inherent in the population of pwMS, the possibility that a real effect on FSS exists was not detected in this pilot.

The results from this study, as with all pilot studies, should be interpreted with caution. The small sample size limits the generalizability of the results, especially given the variability of clinical presentations of pwMS. A follow-up study with a larger sample will be needed to confirm the findings. Stratifying future samples by disease severity with the EDSS might further limit the effects of confounding variables. This study was noncontrolled and nonrandomized, which further limits the generalizations that can be made. The lack of control group significantly limits both internal and external validity of the study; many options for control groups could be used for future studies, including “traditional” nonmaximal strength training (e.g., using 70% of the 1RM), using mobility training as a comparison group for the MST group, and using a no treatment control. The use of the BBS as an outcome measure was somewhat problematic. Many of the participants scored a 53 or above on the BBS pretest limiting the amount of improvement that could have been measured. This ceiling effect could have been reduced by using a different measure of balance such as the Mini-BESTest. Detailed measures of fatigue may clarify whether the identified trend toward increased fatigue may be a concern during MST-based interventions. Collecting mobility measures at 3 and 6 months following the intervention would also be of use, as it will help determine whether the improvements seen after the MST protocol were transitory or sustained. Future studies should also consider including a greater array of LE resistance training, such as knee flexion and ankle plantarflexion, as the use of a more diverse strengthening program may result in greater improvements in functional measures. It may also be of interest to include a mobility measure that captures some of the attributes of power such as the stair climb power test [24]. Such a test will not only expand the clinical implications of MST outcomes but also measure some of the more direct effects of LE resistance training. Future studies of the utility of MST in the MS population would benefit from a study design that incorporates these elements.

## 5. Conclusion

MST is a technique that may be utilized by pwMS to improve strength and mobility. These improvements may occur even though no mobility specific interventions are performed. This may be due to MST resulting in increased neural drive. In this study, MST was not associated with adverse events or increases in fatigue. Larger studies with more sensitive measures will be needed to confirm these findings.

## Figures and Tables

**Table 1:** Participant characteristics.

Age (years), mean (SD)	51.6 years (12.7), range 34–69
EDSS score, median (IQR)	3.0 (1.5), range 2.5–6.0
MS type	
Primary progressive	1
Secondary progressive	4
Relapsing-remitting	2
Years since diagnosis, mean (SD)	14.3 (11.7), range 3–35
MSIS-29	
Physical scale score, mean (SD)	35.2 (15.7), range 16–58
Psychological scale score,	31.7 (17.5), range 3–64
mean (SD)	
Baseline FSS, mean (SD)	44/63 (15.5), range 23/63–60/63

Note: EDSS: Extended Disability Severity Scale; MSIS-29: Multiple Sclerosis Impact Scale-29; FSS, Fatigue Severity Scale.
